# Treatment effects between monotherapy of donepezil versus combination with memantine for Alzheimer disease: A meta-analysis

**DOI:** 10.1371/journal.pone.0183586

**Published:** 2017-08-21

**Authors:** Ruey Chen, Pi-Tuan Chan, Hsin Chu, Yu-Cih Lin, Pi-Chen Chang, Chien-Yu Chen, Kuei-Ru Chou

**Affiliations:** 1 School of Nursing, College of Nursing, Taipei Medical University, Taipei, Taiwan; 2 Department of Nursing, En Chu Kong Hospital, Taipei, Taiwan; 3 Aviation Physiology Research Laboratory, Kaohsiung Armed Forces General Hospital Gangshan Branch, Kaohsiung, Taiwan; 4 Institute of Aerospace and Undersea Medicine, School of Medicine, National Defense Medical Center, Taipei, Taiwan; 5 Department of Neurology, Tri-Service General Hospital, National Defense Medical Center, Taipei, Taiwan; 6 Department of Anesthesiology, Taipei Medical University Hospital, Taipei, Taiwan; 7 Department of Anesthesiology, School of Medicine, College of Medicine, Taipei Medical University, Taipei, Taiwan; 8 Graduate Institute of Humanities in Medicine, Taipei Medical University, Taipei, Taiwan; 9 Department of Nursing, Taipei Medical University-Shuang Ho Hospital, Taipei, Taiwan; 10 Psychiatric Research Center, Taipei Medical University Hospital, Taipei, Taiwan; Banner Alzheimer's Institute, UNITED STATES

## Abstract

**Background:**

This is the first meta-analysis to compare the treatment effects and safety of administering donepezil alone versus a combination of memantine and donepezil to treat patients with moderate to severe Alzheimer Disease, particularly regarding cognitive functions, behavioral and psychological symptoms in dementia (BPSD), and global functions.

**Methods:**

PubMed, Medline, Embase, PsycINFO, and Cochrane databases were used to search for English and non-English articles for inclusion in the meta-analysis to evaluate the effect size and incidence of adverse drug reactions of different treatments.

**Results:**

Compared with patients who received donepezil alone, those who received donepezil in combination with memantine exhibited limited improvements in cognitive functions (g = 0.378, p < .001), BPSD (g = −0.878, p < .001) and global functions (g = −0.585, p = .004). Gradual titration of memantine plus a fixed dose and gradual titration of donepezil as well as a fixed dose and gradual titration of memantine resulted in limited improvements in cognitive functions(g = 0.371, p = .005), BPSD(g = −0.913, p = .001), and global functions(g = −0.371, p = .001).

**Conclusion:**

Both in the 24th week and at the final evaluation point, the combination of donepezil and memantine led to greater improvement in cognitive functions, BPSD, and global functions than did donepezil alone in patients with moderate to severe Alzheimer Disease.

## Introduction

Alzheimer disease (AD) is the most prevalent type of dementia, accounting for more than 80% of cases of dementia in middle- and senior-aged patients. [1] Current treatment strategies primarily focus on medications and are aimed at alleviating symptoms. Cholinesterase inhibitors (ChEIs) and N-methyl D-aspartate (NMDA) receptor antagonists are the two most prevalent types of medicine approved by the U.S. Food and Drug Administration. When the metabolizing enzyme is suppressed, the activity of acetylcholine (Ach) is increased; in turn, cognitive functions improve. [2] In addition, NMDA receptor antagonists regulate glutamatergic neurons activities which facilitate synaptic plasticity, neuronal growth and differentiation, thereby enhancing cognition, learning, and memory.[1, 3] Numerous studies have investigated the treatment effects of the aforementioned medicines on cognitive functions and BPSD in patients with AD.

Patients with moderate to severe AD exhibit relatively severe cognitive and psychological symptoms. ChEIs and NMDA remain the main treatments. Donepezil is the most common ChEI used for AD treatment. Memantine is the most prevalent choice of NMDA. The combination of memantine and donepezil can improve AD symptoms through their different mechanisms. [4–6] Despite the wealth of information on the ChEIs and memantine for treating AD, the magnitude of the effects of administering of donepezil and a combination of memantine and donepezil on patients’ cognitive functions, BPSD, and global functions remains unclear. Therefore, this is the first meta-analysis to compare the effects of administering donepezil alone versus combination of memantine and donepezil for treating patients with moderate to severe AD. We aimed to carry out a scientific and precise meta-analysis with extensive searches from multiple databases to examine: 1) the effect size; 2) moderator analysis; 3) subgroup analysis; and 4) the quality and publication bias on the effect of outcome variables.

## Methods

### Study selection

The databases we searched for this study are from PubMed, PsycINFO, Embase, Ovid Medline, and Cochrane (S1 Table). Our literature search was extended to Google Scholar, since Google Scholar searches literature with a combined ranking algorithm on citation count and keyword relevancy. The selection of articles for this study was limited to peer-reviewed articles. Manual searches were extended to the bibliographies of review articles and included research studies. In order to expand the scope of the search, all summaries, keywords, and full texts were included, and no language restriction was set. We followed the PRISMA statement for reporting systematic reviews and meta-analyses (S2 Table). The final search time was May 2017, with no language restrictions.

### Inclusion and exclusion criteria

All randomized trials were included if they met the following inclusion criteria: (1) studies that focused on patients with diagnosed AD, and (2) studies that compared the effects of administering donepezil (patients who received this treatment are hereafter referred to as the control group) with the combination of memantine and donepezil (those who received this treatment are hereafter referred to as the combination treatment group) on AD treatment, in which the treatment dose of donepezil was 5–10 mg/d. The exclusion criteria of this study were as follows: (1) unrelated to topic, (2) non-relveant population, (3) cell or animals experiment, (4) systematic review or meta-analysis, (5) quantitative research, (6) studies from comment, conference, or letter, (7) non-randomized controlled trial studies, (8) criteria that do not met the inclusion criteria, (9) experiment group combined with other treatment, (10) studies without full-text, (11) duplicate studies on the same sample group form the same author, (12) several outcomes pooled together, and (13) limited data.

### Outcome measures

The results of the effect analysis were divided into main results, secondary results, and subgroups. The main results compared the treatment effects of the control medicine and combination treatment on cognitive functions and BPSD as assessed at the final evaluation point in patients with moderate to severe AD. The secondary results global functions as assessed at the final evaluation point. This study evaluated the incidence of side effects and adverse drug reactions experienced by the two patient groups that occurred in their blood and lymphatic systems, cardiovascular system, central and peripheral nervous systems, digestive system, genitourinary system, mental system, metabolism and nutrition system, musculoskeletal system, nervous system, and respiratory system. In this study, subgroup analyses were performed on intervention characteristics (the combination of donepezil alone vs. memantine and donepezil at 24 weeks) and treatment effects of memantine dose (gradual titration vs. a fixed dose of memantine) on in patients’ cognitive functions, BPSD, and global functions.

### Data extraction

Two investigators (CR, YHL) assessed the relevancy of the search independently. A third investigator (CKR) made the definitive decision for study eligibility and data extraction when discrepancies were found in the inclusion of studies or data extraction.

### Study quality

The selected data and results of all included studies, including the research design, patient demographic data, inclusion and exclusion criteria, dose and duration of medicine application, and the results and side effects of treatment, were analyzed. The Cochrane risk-of-bias tool [7] was applied to assess the quality of each study, and the Jadad quality score [8] was employed as a supplementary assessment tool.

### Additional analyses

In this study, Comprehensive Meta-Analysis Version 2 was used to perform an integrated data analysis. Hedges’ g was used to determine the effect size, and Cohen’s d was used to obtain the overall effect size, with d = 0.1, very small; 0.2, small; 0.5, medium; 0.8, large; 1.2, very large; and d = 2.0, huge. A random effects model was applied [9]. A sensitivity analysis test, namely the I^2^ statistic Q test, was used as the heterogeneity test. Higgins and Thompson [10] proposed the following cutoff values for I^2^ for classifying heterogeneity: I^2^ = 25, low heterogeneity; I^2^ = 50, moderate heterogeneity; and I^2^ = 75, high heterogeneity.

## Results

### Literature search

**Fig 1:**
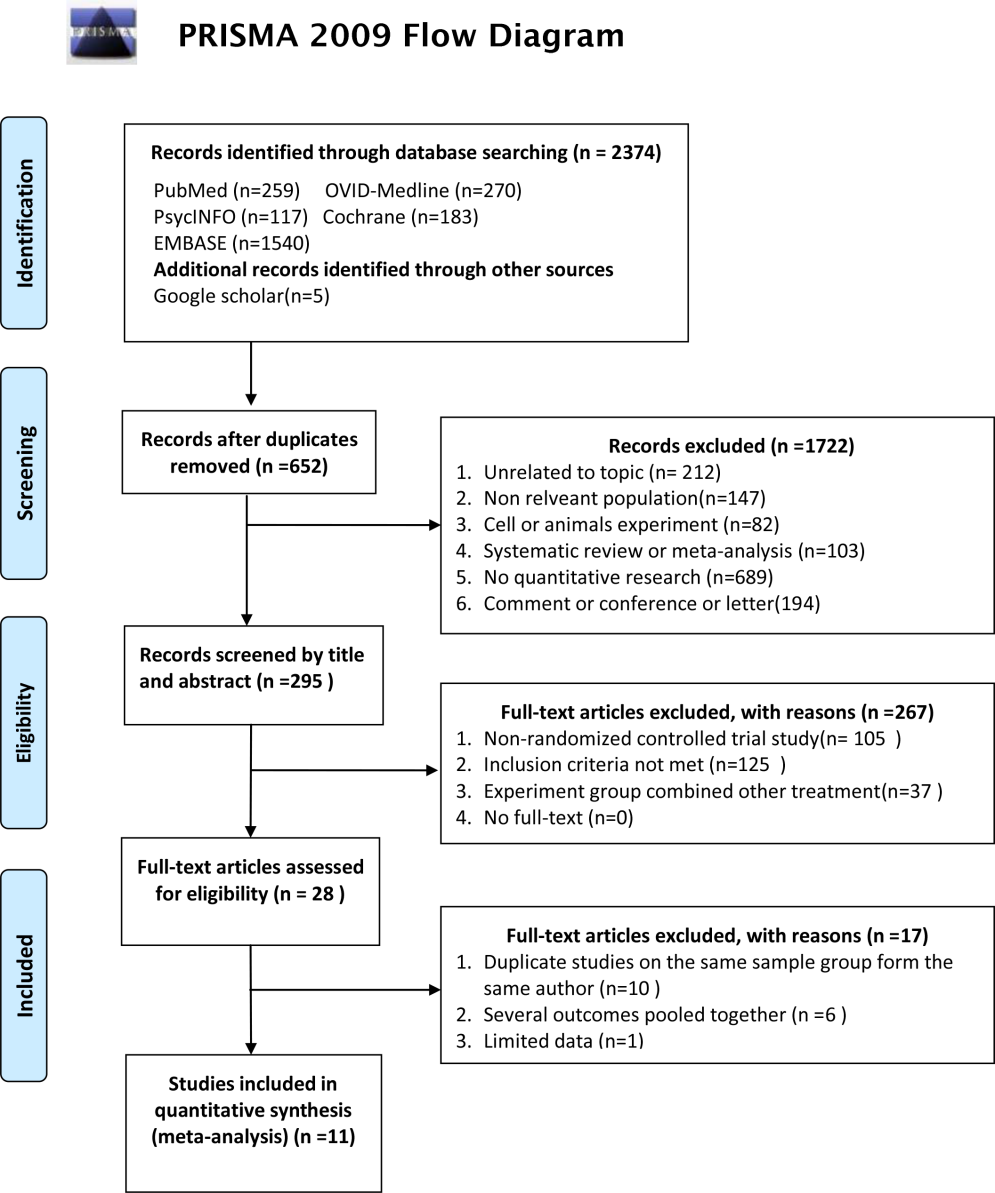
Study selection flow chart.

A total of 2,374 articles were retrieved, of which 652 repeated articles were eliminated. Full-text analysis was then performed, which identified 28 studies that corresponded to the research topic. Of these 28 articles, ten were published repeatedly, six had several outcomes pooled together, and one had limited data. Finally, 11 studies that corresponded to the research topic (2004−2015) were included in the meta-analysis (Fig 1).

### Study characteristics

Table 1[11–21] presents the basic characteristics of the articles included in the present study, as follows: (1) the research periods spanned from 2004 to 2015; (2) by research type, eleven studies were randomized clinical trials. [14]; (3) the diagnosis instruments for AD comprised the Mini-Mental State Examination (MMSE); Diagnostic and Statistical Manual of Mental Disorders, Fourth Edition; Standardized MMSE (SMMSE); and National Institute of Neurological and Communicative Disorders; and (5) sample age ranging from 74.1 to 87.6. The intervention characteristics were as follows: (1) medication application duration spanned from 12 to 52 weeks, and the highest proportion of the studies (five studies) administered medication for 24 weeks [11, 12, 15, 18, 19], and two studies administered medication for 52 weeks[13, 16], and (2) by medication dose, the highest proportion of studies (six studies) administered donepezil incrementally from 5 to 10 mg. In addition, the patient characteristics were as follows: (1) the number of male and female patients was equal; (2) the average patient age ranged from 73.1 to 87.3 years; and (3) the MMSE was the most commonly applied instrument for AD diagnosis.

**Table 1:** Characteristics of the included studies.

Donepezil								Memantine and donepezil						
Study, Year	Country	n	M, %	Age, years, mean ± SD	Severity of AD	Diagnosis Technique (range)	Intervention drug (dose/frequency)	n	M, %	Age, years, mean ± SD	Intervention drug (dose/frequency)	Lost to Follow-up	Data Analysis	Jadad Score / Cochrane tool
Araki 2014	Japan	18	38.9	79.8 ± 4.6	moderate to severe impairment	DSM IV/ ICD10/ HDS-R (3–16)	Donepezil (unclear/ ongoing therapy)	19	57.9	77.9 ± 9.8	Memantine (5 mg/1 wk, 10 mg/2 wk, 15 mg/3 wk, 20 mg/4–24 wk); Donepezil (NA/ongoing therapy)	32.4%	PP	3/ AA: Low AC: Low BAO: Low IO: Low SRO: Low
Doody 2012	USA	303	38.0	74.1 ± 8.7	moderate to severe impairment	MMSE (0–20)	Donepezil (10mg/ongoing therapy 24 wk)	168	36.9	73.1 ± 8.2	Memantine (20 mg/ongoing therapy 24 wk); Donepezil (10 mg/ongoing therapy 24 wk)	18.0%	ITT	4/ AA: Low AC: Low BAO: Low IO: Low SRO: Low
Howard 2012	UK	73	30.1	77.2 ± 7.5	moderate to severe impairment	SMMSE (5–13)	Donepezil (10 mg/ongoing therapy)	73	32.9	77.5 ± 9.0	Memantine (5 mg/1 wk, 10 mg/2 wk, 15 mg/3 wk, 20 mg/4–52 wk); Donepezil (10 mg/ongoing therapy)	23.3%	PP	5/ AA: Low AC: Low BAO: Low IO: Low SRO: Low
Kano 2013	Japan	15	60	76.8 ± 6.2	moderate to severe impairment	NINDS/ADRDA/MMSE (3–14)	Donepezil (10 mg/1–28 wk)	15	53.3	74.4 ± 4.8	Memantine (5 mg/1 wk, 10 mg/2 wk, 15 mg/3 wk, 20 mg/4–28 wk); Donepezil (5 mg/1–28 wk)	9.1%	PP	3/ AA: Low AC: Low BAO: High IO: Low SRO: Low
Mi 2014	China	43	44.2	74.3 ± 6.7	moderate to severe impairment	DSM Ⅳ/ MMSE(≤15)/ HIS (≤4)	Donepezil (5 mg/1–4 wk, 10 mg/ 5–24 wk)	43	32.6	74.0 ± 6.7	Memantine (10 mg/1–24 wk); Donepezil (5 mg/1–4 wk, 10 mg/5–24 wk)	0%	ITT	2/ AA: High AC: High BAO: High IO: Low SRO: Low
Peng 2015	China	38	73.7	82.6 ± 9.6	moderate to severe impairment	NIA-AA/HAMD(≤14) MMSE(≥5)/ HIS(<4)	Donepezil (5 mg/1–52 wk)	38	76.3	83.4 ± 10.1	Memantine (5 mg/1 wk, 10 mg/2 wk, 15 mg/3 wk, 20 mg/4–52 wk); Donepezil (5 mg/1–52 wk)	0%	PP	2/ AA: High AC: High BAO: High IO: High SRO: Low
Shao 2015	China	62	69.4	87.6 ± 2.2	Not reported	DSM /MMSE(10–24)	Donepezil (5 mg/1–16 wk)	62	74.2	87.3 ± 2.1	Memantine (20 mg/1–16 wk); Donepezil (5 mg/1–16 wk)	0%	ITT	3/ AA: Low AC: Low BAO: High IO: Low SRO: Low
Tariot 2004	USA	201	33.3	75.5 ± 8.7	moderate to severe impairment	NINDS/ADRDA MMSE (5–14)/ MRI or CT(AD)	Donepezil (5–10 mg/ongoing therapy)	202	36.6	75.5 ± 8.5	Memantine (5 mg/1 wk, 10 mg/2 wk, 15 mg/3 wk, 20 mg/4–24 wk); Donepezil (5–10 mg/ongoing therapy)	2.2%	ITT	5/ AA: Low AC: Low BAO: Low IO: Low SRO: Low
Wang 2015	China	39	51.3	76.1 ± 6.9	Not reported	DSM/MRI	Donepezil (5 mg/1–4 wk, 10 mg/5–24 wk)	39	53.8	75.5 ± 6.7	Memantine (5 mg/1 wk, 10 mg/2 wk, 15 mg/3 wk, 20 mg/4–24 wk); Donepezil (5 mg/ 1–4 wk, 10 mg/5–24 wk)	0%	ITT	2/ AA: High AC: High BAO: High IO: Low SRO: Low
Yang 2013	China	40	57.5	75.1 ± 1.0	moderate to severe impairment	NINCDS/ADRDA/ MMSE (5–12)/ HIS (≤4)/ BEHAVE-AD (≥8)/CT or MRI (Brain atrophy)	Donepezil (5 mg/1–4 wk, 10 mg/ 5–12 wk)	40	52.5	74.9 ± 1.0	Memantine (5 mg/1 wk, 10 mg/2 wk, 15 mg/3 wk, 20 mg/4–12 wk); Donepezil (5 mg/ 1–4 wk, 10 mg/5–12 wk)	0%	ITT	3/ AA: Low AC: Low BAO: High IO: Low SRO: Low
Zheng 2011	China	16	100	Not reported	Not reported	DSM IV/M MSE (≤ 26)/NCDS -ADRDA/HAMD(≤ 17)/HIS≤4	Donepezil (5 mg/1–4 wk, 10 mg/ 5–16 wk)	16	100	Not reported	Memantine (5 mg/1 wk, 10 mg/2 wk, 15 mg/3 wk, 20 mg/4–16 wk); Donepezil (5 mg/1–4 wk, 10 mg/5–16 wk)	0%	ITT	3/ AA: Low AC: Low BAO: High IO: Low SRO: Low

M, male; SD, standard deviation; DSM IV, Diagnostic and Statistical Manual of Mental Disorders, Fourth Edition; ICD10, International Classification of Diseases, Tenth Edition; Jadad Score: Jadad Score (max = 5); HDS-R(0–30), Hasegawa’s dementia scale-revision; NA, not available; MMSE(0–30), Mini-Mental State Examination; SMMSE(0–30), Standardized Mini-Mental State Examination; NINCDS, National Institute of Neurological and Communicative Disorders, ADRDA, Alzheimer’s Disease and Related Disorders Association; HIS, Hachinski Ischemic Score(0–4); NIA-AA, National Institute of Aging, Alzheimer’s Association; HAMD(0–52), Hamilton Depression Rating Scale; MRI, magnetic resonance imaging, CT, computed tomography; NINDS, National Institute of Neurological and Communicative Disorders and Stroke; BEHAVE-AD(0–78), Behavioral Pathology in Alzheimer Disease; PP, per-protocol; ITT, intention to treat. Cochrane tool: AA = adequacy of sequence allocation; AC = allocation concealment; BAO = blinding of assessors and outcomes; IO = incomplete outcome data; SRO = selective reporting and other biases.

### Quality assessment

According to the assessment results of the Cochrane risk-of-bias tool, four articles exhibited a low risk of bias in all seven items of assessment,[11–13, 18] and none of the included articles reached a high risk of bias in all seven items of assessment. By the domain of bias, 73% of the included articles exhibited low risks of bias for random sequence generation, and 30% exhibited a high risk. Moreover, an equal number of articles exhibited low and high risks of bias for the blinding of patients and personnel; 37% and 63% of the articles exhibited low and high risks of bias for the blinding of outcome assessors, respectively. Furthermore, 90% and 10% of the articles exhibited low and high risks of bias for incomplete outcome data, respectively. All articles exhibited low risk of bias for selective outcome reporting and other biases. According to Jadad quality scores, two articles attained 5 points or more for research quality. [13, 18]

### Primary outcomes

The main results analyzed patient performance in cognitive functions and BPSD at the final evaluation point.

#### Cognitive functions

**Fig 2:**
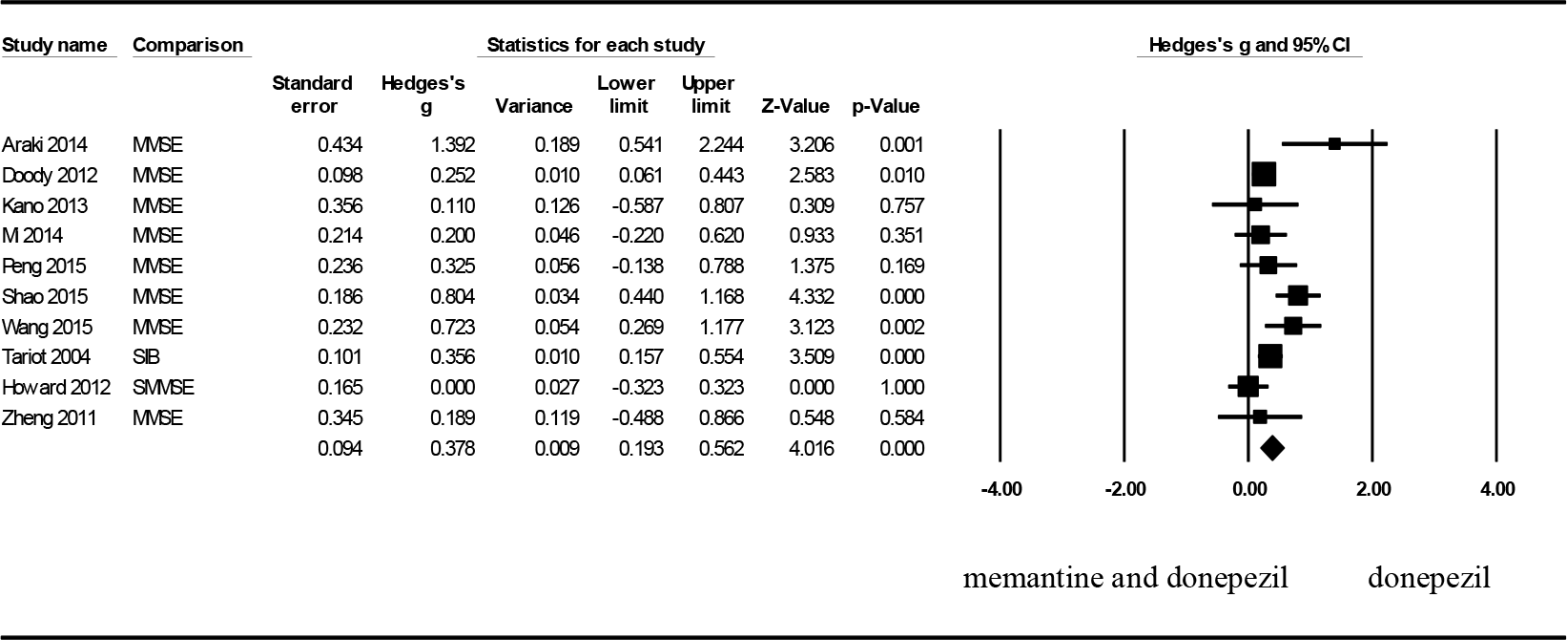
Forest plots to compare the combination therapy with the monotherapy: Cognitive functions.

Regarding the overall cognitive functions observed in the nine articles, the effect size as evaluated using Hedges’ g was 0.378 (95% CI: 0.193–0.562, p < .001, and I^2^ = 57.145), indicating a moderate effect size and significant difference (Fig 2). Consequently, the combination treatment group were more satisfactory than those of patients in the control group. A sensitivity analysis was performed to examine the origin of heterogeneity. The outcome demonstrated that when the article by Shao [17] was removed, Hedges’ g was 0.331 (95% CI: 0.153–0.509 and p = .001), and I^2^ decreased from 56.650 to 43.494, indicating that the origin of heterogeneity may be related to the study by Shao. Moreover, after the results obtained by Shao were removed, a significant difference was still observed between the control group and combination treatment group. Analysis of publication bias showed a symmetrical funnel plot. Egger’s regression test also revealed no publication bias (p = .375).

#### BPSD

**Fig 3:**
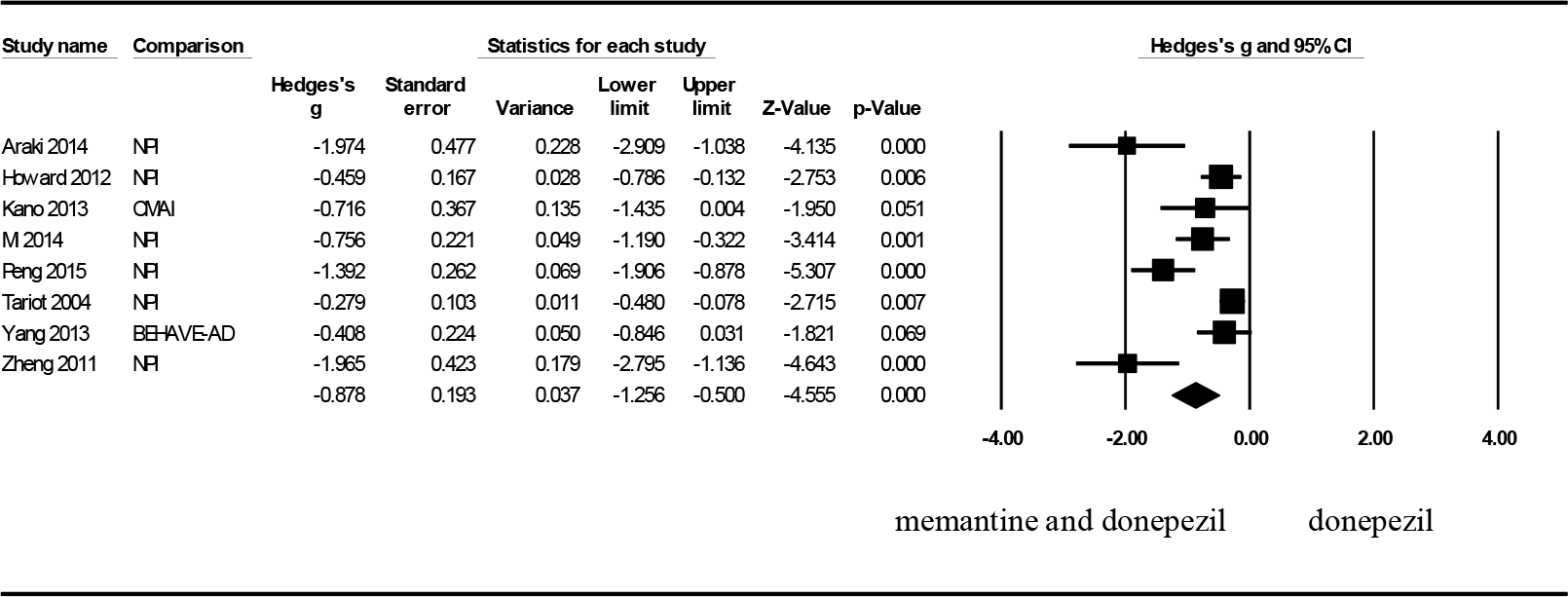
Forest plots to compare the combination therapy with the monotherapy: BPSD.

Eight articles were included in the analysis to determine the treatment effect on BPSD. The effect size of BPSD as expressed through Hedges’ g was –0.878 (95% CI: −1.256 to −0.500, p < .001, and I^2^ = 82.116), achieving a significant difference (Fig 3). Furthermore, patients with moderate to severe AD in the combination treatment group exhibited greater improvements in BPSD than did those in the control group. A sensitivity analysis was subsequently performed to examine the origin of heterogeneity. When the study by Zheng[21] was removed, Hedges’ g was −1.186 (95% CI: −2.127 to −0.245, p = .014), and I^2^ decreased from 81.742 to 77.743, indicating that the origin of heterogeneity may be related to the study by Zheng. Moreover, a significant difference was still observed between the control group and combination treatment group after the study by Zheng was removed. Analysis of publication bias showed an asymmetrical funnel plot. Egger’s regression test also revealed publication bias (p = .005).

### Secondary outcomes

#### Global functions

**Fig 4:**
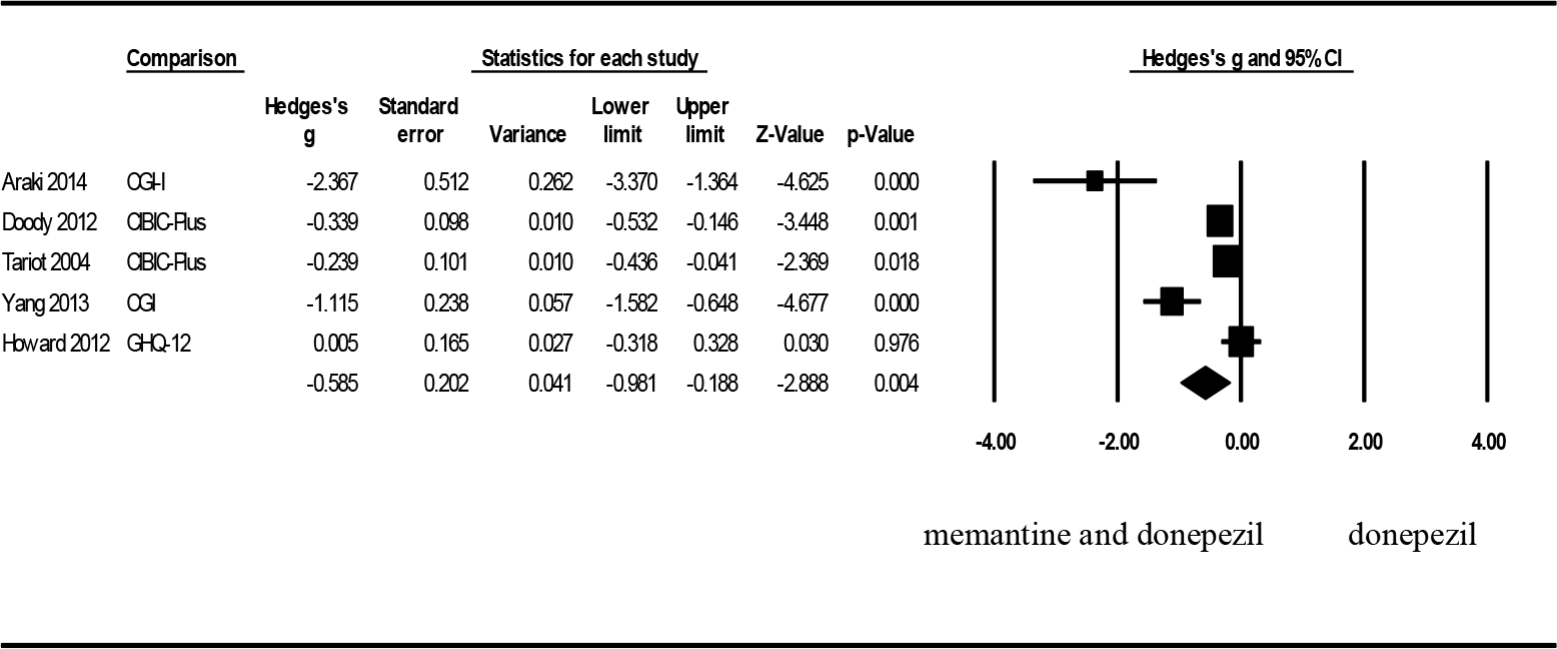
Forest plots to compare the combination therapy with the monotherapy: global functions.

Five articles were included in the current analysis for overall functional evaluations. The effect size as expressed through Hedges’ g was −0.585 (95% CI: −0.981 to −0.188, p = .004, and I^2^ = 87.358), achieving a significant difference (Fig 4). Consequently, patients with moderate to severe AD in the combination treatment group exhibited greater improvement in global functions than did those in the control group. The heterogeneity test revealed a high level of heterogeneity. A sensitivity analysis was conducted to investigate the origin of heterogeneity on the basis of the medication application duration. Further analysis of the sample sizes and doses of donepezil yielded a Hedges’ g of −0.629 (95% CI: −1.208–0.049, p = .0034, and I^2^ = 89.448). When articles were eliminated individually, I^2^ remained within the range of 85.577–91.869, showing no tendency of decreasing. Analysis of publication bias showed a symmetrical funnel plot. Egger’s regression test also revealed no publication bias (p = .123).

### Subgroup analysis

In the 24th week, a comparison of the combination treatment group and control group showed significant differences in cognitive functions, BPSD, and global functional evaluation (Table 2). In the combination treatment group, the gradual titration and fixed dose of memantine led to significant improvements in cognitive functions, BPSD, and global functions.

**Table 2:** Subgroup analysis results of study outcomes.

	Cognition Function					Behavioral and Psychological Symptoms in Dementia					Global Functions				
	No.of Trials	Hedges’ g (95% CI)	*P* value	Overall *P* value	*I*^2^	No.of Trials	Hedges’ g(95% CI)	*P* value	Overall *P* value	*I*^2^	No.of Trials	Hedges’ g(95% CI)	*P* value	Overall *P* value	*I*^2^
Intervention characteristics															
Combination of donepezil alone vs. memantine and donepezil at 24 weeks	6	0.391 (0.180–0.603)	0.001		50.981	4	−0.767 (−1.314 to −0.219)	.006		79.920	3	−0.583 (−1.145 to −0.021)	.042		91.081
Treatment effects of memantine dose				.864	57.15				.068	82.12				0.001**	89.488
Gradual Titration (5–20 mg)	7	0.371 (0.111–0.631)	0.005			7	−0.913 (−1.349 to −0.476)	.001			4	−0.371 (−0.676 to −0.066)	.001		
Fix Dose(10/20 mg)	3	0.408 (0.178–0.591)	0.018			1	−0.756 (−1.85 to −0.339)	.176			1	−2.367 (−3.503 to −1.231)	.069		

CI = confidence interval

**P ≤ .001.

#### Adverse events

Adverse drug reactions observed in the two groups were compared using 14 items, namely the adverse reactions in 12 systems, other adverse reactions, and death. The highest frequency of adverse drug reactions occurred in the digestive system, comprising a total of 23 events and exhibiting an RR of 0.889 (95% CI: 0.621–1.274, p = .522, and I^2^ = 3.216). The second highest number of adverse drug reactions occurred in the mental system, comprising to a total of 10 events and exhibiting an RR of 1.501 (95% CI: 0.932–2.417, p = .095, and I^2^ = 15.143). The most severe adverse drug reaction was death; a total of two deaths were observed, exhibiting an RR of 0.521 (95% CI: 0.227–1.195, p = .550, and I^2^ = 0.001) (Table 3). No significant statistical difference was observed for the 14 items of adverse drug reactions (RR = 1.079, 95% CI: 0.925–1.259, p = .330, and I^2^ = 0.001) between the combination treatment group and control group, indicating that the medicines administered to these two groups resulted in no significant difference in safety or adverse drug reactions.

**Table 3:** Adverse event and risk ratio of the combination treatment primary outcomes in Alzheimer disease (endpoint).

	N	Effect Sizes	Null Hypothesis (2-tail)		Heterogeneity(*P* >.10)			
Adverse Event		Hedges’ g/Risk Ratio(95% CI)	Z value	*P* value	Q value	P value	*I*^2^	Tau^2^
Digestive system	23	0.889 (0.621–1.274)	−0.641	.522	22.731	.417	3.216	0.159
Mental system	10	1.501 (0.932–2.417)	1.671	.095	10.606	.304	15.143	0.296
Central and peripheral nervous systems	7	1.153 (0.739–1.798)	0.629	.530	6.299	.391	4.748	0.139
Cardiovascular system	6	1.485 (0.566–3.893)	0.803	.422	6.651	.248	24.827	0.597
Genitourinary system	6	1.271 (0.764–2.113)	0.923	.356	0.943	.967	0.000	0.000
Musculoskeletal system	6	1.808 (0.423–7.726)	0.799	.424	6.492	.261	22.980	0.489
Systemic	5	1.235 (0.667–2.286)	0.671	.502	1.814	.770	0.001	0.001
Respiratory system	4	0.886 (0.432–1.816)	−0.330	.741	3.458	.326	13.236	0.287
Metabolism and nutrition systems	3	1.225 (0.647–2.320)	0.622	.534	0.562	.755	0.001	0.001
Nervous system	3	1.808 (0.645–3.420)	0.929	.353	4.826	.090	58.562	0.962
Death	2	0.521 (0.227–1.195)	−1.539	.124	0.081	.776	0.001	0.001
Blood and lymphatic systems	2	1.345 (0.141–12.829)	0.257	.797	0.489	.484	0.001	0.001
Other	2	0.691 (0.334–1.430)	−0.996	.319	0.808	.369	0.001	0.001
Cancer	1	0.200 (0.010–4.095)	−1.045	.296	0.001	1.000	0.001	0.001

CI = confidence interval

### Meta-regression analysis

We performed a meta-regression analysis to identify the potential moderating variables. The results revealed no significant difference in the medication application duration (p = .068–.785).

## Discussion

The most crucial finding of the present study was that at the endpoint or in the 24th week of treatment for moderate to severe AD, the combination treatment group exhibited greater improvement in cognitive functions, BPSD, and global functions than did the control group. No significant difference was observed in adverse drug reactions, safety between the two groups.

Memantine and donezepil exhibit different mechanisms of action for AD. Other systematic studies have also indicated that when the course of the disease progresses to moderate or severe levels, combination treatments are more effective than single treatment for delaying the degradation of cognitive functions. [4, 5, 13, 22–26] This finding is consistent with that of the present meta-analysis.

Regarding the effect of treatments on cognitive functions, the finding of the present study was significant but exhibited moderate heterogeneity. A sensitivity analysis was conducted at the final evaluation point. Compared with other studies, Shao [17] included a different medication dosage (memantine, 20 mg/1–16 wk; donepezil, 5 mg/1–16 wk) and different disease severity. The present study also identified that in Week 24, the effect size differed significantly between the combination treatment group and control group, indicating that in Week 24, the combination treatment group exhibited greater improvement in cognitive functions than did the control group. Nevertheless, a moderate level of heterogeneity remained.

Theoretically, donepezil can mitigate BPSD. Research has identified donepezil as a second-line treatment for behavioral and psychological symptoms such as apathy, depression, and aberrant motor behavior. [27, 28] However, several meta-analyses have revealed that donepezil exerts limited effects for improving BPSD. [22, 29] A part of the brain cortex and the neurons in the hippocampus that synthesize the excitatory amino acid glutamic acid (glutamate) are related to human memory function. Memantine is a glutamatergic NMDA receptor antagonist that protects neural cells from overstimulation by glutamate, thereby lowering the excitotoxicity of glutamate. In addition to suppressing cognition impairment, memantine can be used as a second-line treatment to prevent aimless wondering, stereotypic behaviors, irritability, and aggressiveness. [18, 30, 31] Previous meta-analyses have revealed that the treatment effect of memantine on BPSD is not significant [32]. Recent studies have used the combination of memantine and donepezil to treat the BPSD of patients with AD, because the two medicines focus on different types of BPSD, and the pharmacological mechanisms and the targets of these two medicines differ. [11, 23, 31] Several studies have demonstrated that compared with single-medicine treatments, the combination treatment is more effective for improving and mitigating BPSD. [4, 5, 24–26] The results of the present meta-analysis indicate that the use of the studied medicine combination resulted in a larger effect size than the use of a ChEI alone in BPSD treatment. Consequently, the optimal treatment effect on BPSD can be achieved through the combination of the two studied medicines.

Global assessment is a means of measuring the clinical relevance of any improvement in cognitive functions. The combination treatment also improved global functions, and the effect of the combination treatment was more satisfactory than that of donepezil alone. [3, 4, 25, 26] The present meta-analysis also identified a large effect size and high heterogeneity at both the final evaluation point and in Week 24, confirming that the combination treatment exerted a more satisfactory effect than donepezil alone.

Common adverse drug reactions occur when donepezil is administered to treat AD, including those in the digestive system (e.g., nausea, vomiting, and diarrhea), psychological system (e.g., irritability and anxiety), and bradycardia. [3, 4, 22] When memantine was administered to treat AD, the adverse drug reactions mostly occurred in the digestive system (e.g., nausea, diarrhea, and constipation), followed by the psychological system (i.e., confusion and excitement).[33, 34] When the combination of memantine and donepezil was used to treat AD, no difference was observed in the incidence of adverse reactions between the combination treatment group and control group, demonstrating that the combination treatment does not exhibit a higher rate of adverse drug reactions than donepezil alone.

## Conclusions

The results showed that for treating patients with moderate to severe AD, the combination therapy limited superiority more than donepezil alone for improving cognitive functions, BPSD, and global functions. By contrast, no significant difference was observed in drug safety between the two groups. The strength of this study is that the data analysis was based on the sources from multiple languages rather than English-only sources, and the methodology was effectively designed. The limitation of this study was the heterogeneity across studies, and the sample size varied among the investigated studies. The conclusions were still limited because of the small sample size. Future studies should consider performing large-scale randomized controlled trials or prospective cohort studies in which intervention measures and assessment instruments are combined to control individual variability and latent disturbing factors; this would confirm whether the combination of memantine and donepezil is more effective than donepezil alone for treating patients with moderate to severe AD.

## Supporting information

S1 Table Search strings by database. (DOCX)

S2 Table PRISMA 2009 checklist. (DOC)
