# Supplementary material for: Treatment effects between monotherapy of donepezil versus combination with memantine for Alzheimer disease: A meta-analysis
Source: PLoS One. 2017 Aug 21;12(8):e0183586. doi: 10.1371/journal.pone.0183586 (PMC5565113; doi:10.1371/journal.pone.0183586)
Supplement: S1 Table — (DOCX) [file pone.0183586.s001.docx]

**S1 Table. Search strings by database**

| **PubMed** | | | |
| --- | --- | --- | --- |
| Group | Search terms |  | Exact dates of search |
| 1# | Search Memantine |  | 2017/5/26 |
| 2# | Search Donepezil |  |  |
| 3# | Search Alzheimer Disease[MeSh Terms] |  |  |
| 1# AND 2# AND 3# |  | 259 |  |
| **PsycINFO** | | | |
| Group | Search terms |  | Exact dates of search |
| 1# | Memantine.mp |  | 2017/5/26 |
| 2# | Donepezil.mp |  |  |
| 3# | Alzheimer disease.mp |  |  |
| 1# AND 2# AND 3# |  | 117 |  |
| **EMBASE** | | | |
| Group | Search terms |  | Exact dates of search |
| 1# | memantine' |  | 2017/5/26 |
| 2# | donepezil' |  |  |
| 3# | 'alzheimer disease'/mj |  |  |
| 1# AND 2# AND 3# |  | 1540 |  |
| **Medline** | | | |
| Group | Search terms |  | Exact dates of search |
| 1# | memantine |  | 2017/5/26 |
| 2# | donepezil |  |  |
| 3# | Alzheimer Disease |  |  |
| 1# AND 2# AND 3# |  | 270 |  |
| **Cochrane** | | | |
| Group | Search terms |  | Exact dates of search |
| 1# | memantine |  | 2017/5/26 |
| 2# | donepezil |  |  |
| 3# | Alzheimer disease | 183 |  |
